# Long noncoding RNA SNHG25 promotes the malignancy of endometrial cancer by sponging microRNA-497-5p and increasing FASN expression

**DOI:** 10.1186/s13048-021-00906-w

**Published:** 2021-11-18

**Authors:** Yuhua He, Shuifang Xu, Yi Qi, Jinfang Tian, Fengying Xu

**Affiliations:** Department of Gynaecology and Obstetrics, Jinshan District Tinglin Hospital, 80 North Siping Road, Jinshan District, Shanghai, 201505 China

**Keywords:** SNHG25, Endometrial cancer, ceRNA, miRNA sponge

## Abstract

**Background:**

Small nucleolar RNA host gene 25 (SNHG25), a long noncoding RNA, has been well-studied in epithelial ovarian cancer. However, the specific functions of SNHG25 in endometrial cancer (EC) have not been studied yet. In this study, we aimed to elucidate the clinical significance of SNHG25 in EC and determine the regulatory activity of SNHG25 on the tumor-associated EC phenotype. We also thoroughly explored the molecular mechanisms underlying SNHG25 function in EC.

**Methods:**

Gene expression was measured using quantitative real-time polymerase chain reaction. The detailed functions of SNHG25 in EC were examined by performing loss-of-function experiments. Moreover, the regulatory mechanisms involving SNHG25, microRNA-497-5p, and fatty acid synthase (FASN) were unveiled using the luciferase reporter assay and RNA immunoprecipitation.

**Results:**

We observed a high level of SNHG25 in EC using the TCGA dataset and our study cohort. Patients with a high SNHG25 level had shorter overall survival than those with a low SNHG25 level. SNHG25 deficiency resulted in tumor-repressing activities in EC cells by decreasing cell proliferation, migration, and invasion and promoting cell apoptosis. Furthermore, the function of SNHG25 depletion in impairing tumor growth in vivo was confirmed. SNHG25 sequestered miR-497-5p as a competing endogenous RNA in EC and consequently positively regulated FASN expression. Thus, the decrease in miR-497-5p or increase in FASN could neutralize the modulatory actions of SNHG25 knockdown in EC cells.

**Conclusions:**

The depletion of SNHG25 impedes the oncogenicity of EC by targeting the miR-497-5p/FASN axis. The newly elucidated SNHG25/miR-497-5p/FASN pathway may be a promising target for the molecular-targeted management of EC.

## Background

Endometrial cancer (EC) is the fourth most common gynecological tumor among women worldwide [1]. Recently, the morbidity of EC has shown a gradually ascending trend, and EC is being diagnosed at a young age [2]. Approximately 380,000 EC cases are recorded annually, with 89,000 mortalities globally [3]. At present, owing to the substantial improvements in therapeutic techniques, patients with EC usually achieve favorable outcomes, with the 5-year survival rate of approximately 85% [4]. However, a large number of patients with EC are diagnosed at the advanced stage at the time of symptom onset, and these therapeutic interventions are not effective for such patients, resulting in the 5-year survival rate to decrease to < 20% [5]. Many risk factors have been implicated in EC pathogenesis; however, the principal molecular events occurring downstream of this disease remain elusive [6]. Thus, more knowledge of EC onset and progression at the molecular level may play a key role in devising treatment strategies for EC and consequently achieving better therapeutic efficacy.

Genetic studies have illustrated that noncoding RNAs account for the vast majority of the human genome [7]. Long noncoding RNAs (lncRNA) constitute a group of noncoding transcripts whose length is > 200 nucleotides [8]. Although these are not translated into protein, they play crucial roles in various human disorders [9]. Currently, the dysregulation of lncRNAs is implicated in almost all human cancer types, and lncRNAs have been reported to act as cancer promoters or inhibitors [10]. Notably, a considerable number of studies have proved that accumulated lncRNAs are differentially expressed in EC and participate in diverse malignant processes during endometrial carcinogenesis and progression [11–13].

MicroRNAs (miRNAs) are 17–23 nucleotide long RNA transcripts without protein-coding capacity [14]. They work via base pairing to the 3′-untranslated regions (3′-UTRs) of their target genes and consequently result in translational suppression or mRNA degradation [15]. miRNAs execute tumor-promoting or tumor-repressing roles during oncogenesis and cancer progression by affecting tumor-associated genes [16]. Importantly, lncRNAs are capable of competitively binding to miRNAs and thereby separating miRNA from their targets, consequently giving rise to the competing endogenous RNA (ceRNA) theory [17]. Therefore, researching lncRNAs, miRNAs, and relevant working pathways may aid in the management of EC.

Small nucleolar RNA host gene 25 (SNHG25) has been well-studied in epithelial ovarian cancer [18]. Nevertheless, the specific functions of SNHG25 in EC have not been studied. In this study, we aimed to elucidate the clinical significance of SNHG25 in EC and determine the regulatory activities of SNHG25 on the tumor-associated EC phenotype. We also thoroughly explored the molecular mechanisms underlying SNHG25 function in EC. Our findings may provide a theoretical foundation for developing novel biomarkers for EC treatment.

## Methods

### Clinical samples and cell lines

This study was approved by the Ethics Committee of Jinshan District Tinglin Hospital. After obtaining signed informed consent, 46 pairs of EC and adjacent normal tissues of patients with EC were collected from the hospital. None of the patients had undergone preoperative hormone therapy, radiotherapy, or chemotherapy. The tissues were preserved in liquid nitrogen until use.

Four EC cell lines were used: HEC-1-B and AN3CA were grown in Minimum Essential Medium (Gibco, Thermo Fisher Scientific, Inc., Waltham, MA, USA), whereas RL95–2 and KLE were grown in DMEM:F12 medium (Gibco). All aforementioned cell lines were acquired from the American Type Culture Collection (Manassas, VA, USA). Further, 10% fetal bovine serum (FBS) and 1% penicillin/streptomycin (Gibco) were added to the culture medium. Human endometrial epithelial cells were cultured in a complete culture medium for human endometrial epithelial cells (both from Procell Life Science & Technology Co., Ltd., Wuhan, China). All cells were maintained in a humidified atmosphere with 5% CO_2_ at 37 °C.

### Transfection assay

Shanghai GenePharma Co., Ltd. (Shanghai, China) designed and synthesized the small interfering RNA (siRNA)-targeted SNHG25 (si-SNHG25) and negative control (NC) siRNA (si-NC). The si-SNHG25#1 sequence was 5′-ATCTATCACTCTCGTTCTTGTAG-3′, the si-SNHG25#2 sequence was 5′-TTCCGGGAGGTCAGGTTGTATTC-3′, the si-SNHG25#3 sequence was 5′-GACATTCCAATTAAAGCACGTGT-3′ and the si-NC sequence was 5′-CACGATAAGACAATGTATTT-3′. FASN overexpression plasmid pcDNA3.1-FASN was prepared by GenScript Biotech Corp (Nanjing, China). The miRNA oligonucleotides, including miR-497-5p mimic, NC mimic, miR-497-5p inhibitor (anti-miR-497-5p), and NC inhibitor (anti-NC), were also acquired from Shanghai GenePharma Co., Ltd. The sequences used were: miR-497-5p mimic, 5′-UGUUUGGUGUCACACGACGAC-3′; NC mimic, 5′-UUGUACUACACAAAAGUACUG-3′; anti-miR-497-5p, 5′-ACAAACCACAGUGUGCUGCUG-3′; and anti-NC, 5′-ACUACUGAGUGACAGUAGA-3′. The transfection experiment was conducted using Lipofectamine® 2000 (Invitrogen, Thermo Fisher Scientific, Inc.).

### Quantitative real-time polymerase chain reaction (qRT-PCR)

Total RNA extraction was performed using TRIzol reagent (Invitrogen, Carlsbad, CA, USA). Total RNA was reverse transcribed into complementary DNA (cDNA) using PrimeScript™ RT reagent Kit (Takara, Dalian, China). Subsequently, TB Green® Premix Ex Taq™ II (Takara) was used for measuring SNHG25 and FASN expression. GAPDH acted as the reference control for SNHG25 and FASN expression. For determining miR-497-5p expression, miRNA-specific cDNA was synthesized using Mir-X miRNA First-Strand Synthesis Kit. The cDNA was then subjected to polymerase chain reaction (PCR) with Mir-X miRNA quantitative real-time PCR (qRT-PCR) TB Green® Kit (Takara). Small nuclear RNA U6 served as the reference gene for miR-497-5p. The 2^−ΔΔCq^ method was applied for gene expression calculation.

The sequences of the primers were: SNHG25 forward, 5′-GTTCCGGGAGGTCAGGTTGTA-3′ and reverse, 5′-GCTCAGACTCCAGTTCGCATC-3′; FASN forward, 5′-TCATCCGCTCGTTGTACCAGT-3′ and reverse, 5′-TGGACTTGGTGGAGCCGAT-3′; GAPDH forward, 5′-AGTCAACGGATTTGGTCGTATTG-3′ and reverse, 5′-AAACCATGTAGTTGAGGTCAATGAA-3′; miR-497-5p forward, 5′-TCGGCAGGCAGCAGCACACUG-3′ and reverse, 5′-CACTCAACTGGTGTCGTGGA-3′; and U6 forward, 5′-CTCGCTTCGGCAGCACA-3′ and reverse, 5′-AACGCTTCACGAATTTGCGT-3′.

### Subcellular fractionation experiment

The nuclear and cytoplasmic fractions of EC cells were separated using PARIS kit (Thermo Fisher Scientific, Inc.). The RNA from the nuclear and cytoplasmic fractions was extracted and then analyzed via qRT-PCR for assessing relative SNHG25 distribution.

### Cell counting Kit-8 assay

The cell suspension was prepared by mixing 2 × 10^3^ cells with 1 mL of complete culture medium. Each well of the 96-well plate was covered with 100 μL of cell suspension, followed by culturing for different time periods. Subsequently, the cells were incubated with the Cell Counting Kit-8 (CCK-8) reagent (Beyotime, Shanghai, China) at 37 °C for 2 h. Finally, the absorbance at 450 nm wavelength was measured using a microplate reader.

### Transwell migration and invasion experiments

For migration experiments, the cell suspension was prepared by adding 5 × 10^5^ cells into 1 mL of FBS-free culture medium. The upper chambers of Transwell inserts (BD Biosciences) were covered with 200 μL of the suspension. Complete culture medium supplemented with 20% FBS was added into the lower chambers. After culturing for 24 h, the nonmigrated cells were cleaned by wiping the upper layer of the membrane with a cotton bud. Thereafter, 4% paraformaldehyde was applied for fixing the migrated cells, followed by staining using 0.5% crystal violet. The experimental procedures of the invasion experiments were similar to the aforementioned procedures, with the exception that the membranes were coated with Matrigel (BD Biosciences) before cell seeding. An inverted microscope (Leica, Wetzlar, Germany) was used to acquire cell images and count the migrated/invaded cells in five randomly selected fields.

### Flow cytometry analysis

Annexin V-FITC Apoptosis Detection Kit (Beyotime, Shanghai, China) was used for assessing cell apoptosis. Transfected cells were harvested via trypsin treatment and centrifuged after being washed with phosphate-buffered saline. The cells were then resuspended in 195 μL of Annexin V-FITC buffer and subsequently stained in the dark using 5 μL of Annexin V-FITC and 10 μL of propidium iodide at 20 °C for 30 min. The apoptotic cells were then differentiated and analyzed using a flow cytometer (BD Biosciences, Franklin Lakes, NJ, USA).

### Tumor xenografts in nude mice

The animal experiment was conducted with the approval of the Animal Ethics Committee of Jinshan District Tinglin Hospital. Short-hairpin RNA (shRNA) targeting SNHG25 (sh-SNHG25) and NC shRNA (sh-NC) were synthesized by Shanghai GenePharma Co., Ltd. The sh-SNHG25 sequence was 5′-CCGGTTCCGGGAGGTCAGGTTGTATTCCTCGAGGAATACAACCTGACCTCCCGGAATTTTTG-3′ and the sh-NC sequence was 5′-CCGGCACGATAAGACAATGTATTTCTCGAGAAATACATTGTCTTATCGTGTTTTTG-3′.The shRNAs were inserted into the pLKO.1 plasmid before being transfected into 293 T cells in parallel with psPAX2 packaging plasmid and pMD2.G envelope plasmid. After the cells were cultured at 37 °C with 5% CO_2_ for 48 h, the lentiviruses stably expressing sh-SNHG25 or sh-NC were harvested and used to infect HEC-1-B cells. The stably transfected cells were screened using puromycin.

Female BALB/c nude mice (Vital River Laboratory Animal Technology Co., Ltd., Beijing, China), 4–6 weeks old, were randomly classified into two groups, and injected with HEC-1-B cells with stable sh-SNHG25 or sh-NC transfection, respectively. Tumor width and length were measured once every 5 days to calculate the tumor volume. The tumor volume was calculated using the following formula: volume (mm^3^) = 0.5 × length (mm) × width^2^ (mm^2^). All mice were euthanized by cervical dislocation at 30 days post cell injection. Subcutaneous xenografts were removed from nude mice and weighted.

### Bioinformatic analysis

The downstream targets of SNHG25 were predicted using starBase version 3.0 (http://starbase.sysu.edu.cn). The potential targets of miR-497-5p were searched using TargetScan (http://www.targetscan.org/), miRDB (http://mirdb.org/), and starBase version 3.0.

### RNA immunoprecipitation

RNA immunoprecipitation (RIP) was performed using EZ-Magna RIP™ RNA-Binding Protein Immunoprecipitation Kit (EMD Millipore, Billerica, MA, USA) to examine the binding among SNHG25, miR-497-5p, and FASN. Cells were treated with RIP lysis buffer and centrifuged at 1000×*g* at 4 °C for 15 min. The obtained cell lysate was cultured overnight with magnetic beads conjugated with anti-Argonaute 2 (Ago2) or IgG antibody (EMD Millipore) at 4 °C. After detachment of the cells using protease K, the immunoprecipitated RNA was extracted and examined using qRT-PCR.

### Luciferase reporter assay

SNHG25 and FASN sequences containing predicted wild-type (wt) miR-497-5p binding site were amplified by Shanghai GenePharma Co., Ltd., and inserted downstream of the luciferase reporter plasmid pmirGLO (Promega, Madison, WI, USA). The produced luciferase reporter plasmids were named SNHG25-wt and FASN-wt, respectively. The luciferase reporter plasmids harboring the mutant (mut) binding site, namely SNHG25-mut and FASN-mut, were prepared employing similar experimental procedures. After seeding into 24-well plates, wt or mut reporter plasmids in combination with miR-497-5p mimic or NC mimic were cotransfected into EC cells, followed by culturing at 37 °C for 48 h. The activity triggered by luciferase reporter plasmids was measured using a dual-luciferase reporter system (Promega).

### Western blotting

Following total protein extraction using RIPA buffer, total protein quantification was performed using BCA Protein Assay Kit (both from Beyotime). The same amount of protein was separated by sodium dodecyl sulfate/polyacrylamide gel electrophoresis. Subsequently, the resolved proteins were transferred onto polyvinylidene difluoride membranes and then blocked with 5% fat-free milk at room temperature for 2 h. Next, the membranes were incubated with primary antibodies, anti-FASN (ab128870) and anti-GAPDH (ab181603; Abcam, Cambridge, UK), at 4 °C overnight. Following incubation with the secondary antibody (ab205718; Abcam) at room temperature for 1 h, the membranes were visualized using an Enhanced Chemiluminescence Kit (Pierce; Thermo Fisher Scientific, Inc.).

### Statistical analysis

All data were expressed as mean ± standard deviation. Comparison of data between the two groups was performed using the Student’s *t*-test. One-way analysis of variance followed by Tukey’s test was employed to confirm the differences among multiple groups. The expression relationship was examined via Pearson’s correlation analysis. The Kaplan–Meier method was used for survival analysis, and the overall survival curves were compared using the log-rank test. *P* < 0.05 was considered statistically significant.

## Results

### Loss of SNHG25 results in anticarcinogenic activities in EC cells

**Fig. 1:**
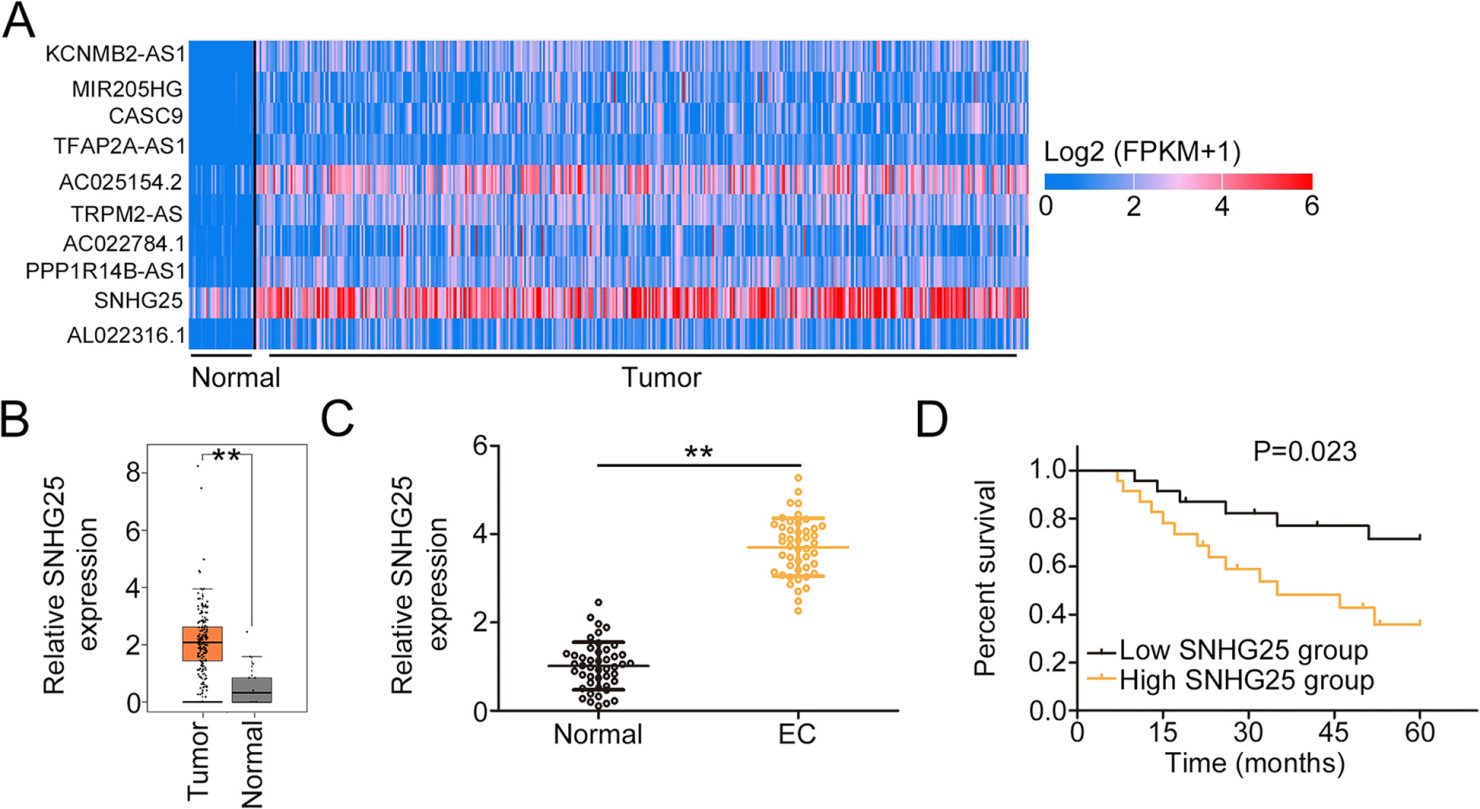
Increased SNHG25 level was confirmed in EC. **A** The top 10 overexpressed lncRNAs in uterine corpus endometrial carcinoma. **B** The expression of SNHG25 in uterine corpus endometrial carcinoma was examined using the TCGA database. **C** qRT-PCR was used for determining the expression of SNHG25 in EC tissues. **D** The relationship between SNHG25 level and overall survival in patients with EC was examined using the Kaplan–Meier curve. **P < 0.01

Through the TCGA database, SNHG25 was found to be one of the top 10 overexpressed lncRNAs in uterine corpus endometrial carcinoma (Fig. 1A). The SNHG25 level was increased in uterine corpus endometrial carcinoma tissues compared with normal tissues (Fig. 1B). Furthermore, a striking upregulation of SNHG25 was observed in EC tissues compared with adjacent normal tissues (Fig. 1C). A high level of SHNG25 was associated with a notably shorter overall survival (Fig. 1D).

**Fig. 2:**
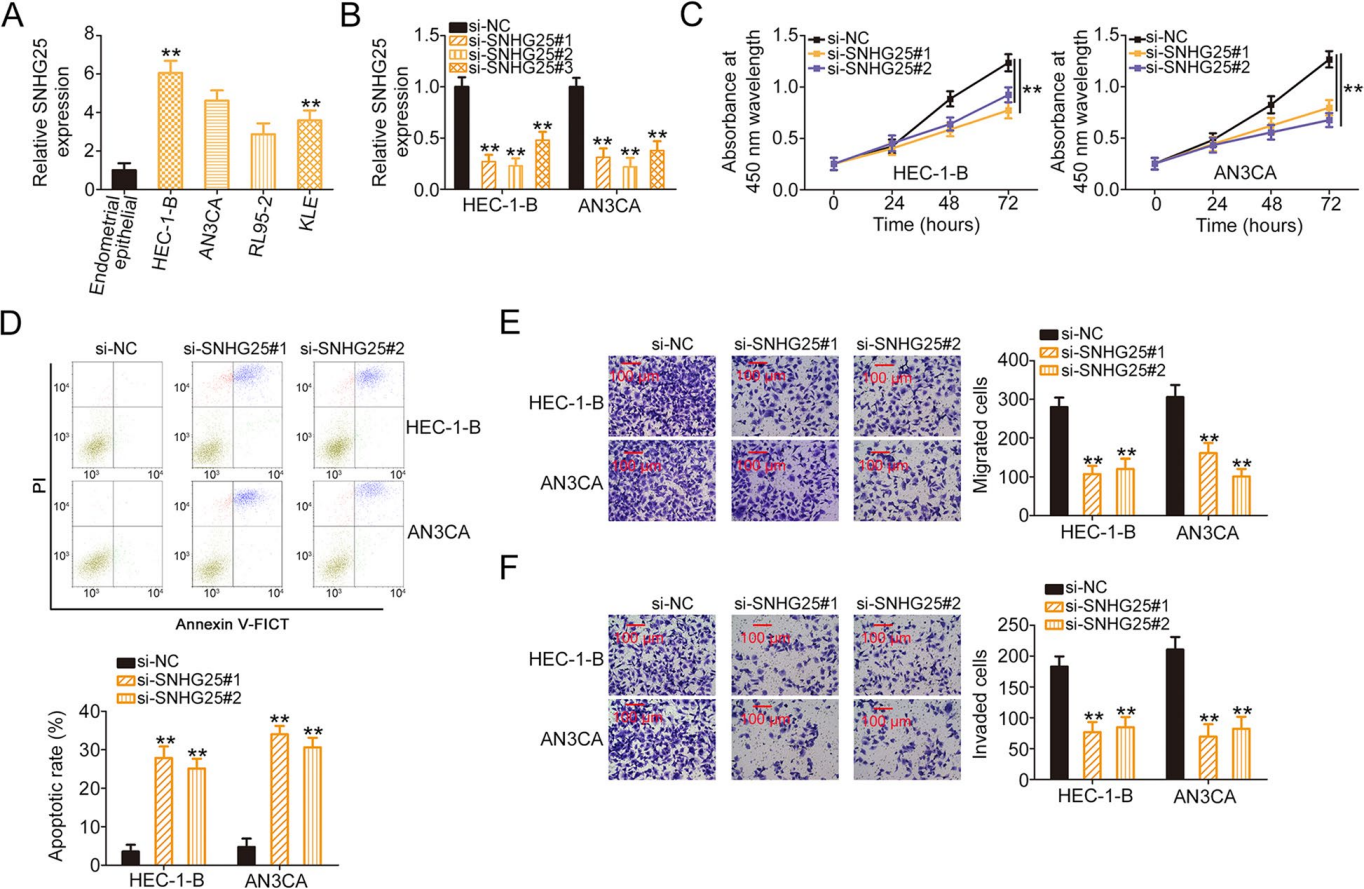
SNHG25 interference hampered the biological characteristics of EC cells. **A** SNHG25 expression in EC cell lines. **B** qRT-PCR was used for determining the expression of SNHG25 in HEC-1-B and AN3CA cells overexpressing si-SNHG25. **C** CCK-8 assay was used to evaluate the proliferation of EC cells expressing si-SNHG25. **D** The proportion of apoptotic cells among EC cells with SNHG25 silencing was quantified. **E**, **F** The migratory and invasive properties of SNHG25-depleted EC cells were examined using the Transwell migration and invasion assays. **P < 0.01.

We next attempted to elucidate the effects of SHNG25 in controlling the malignant behaviors of EC cells. The four EC cell lines showed distinct SNHG25 overexpression, particularly HEC-1-B and AN3CA (Fig. 2A). Therefore, HEC-1-B and AN3CA cell lines were used in subsequent experiments. Moreover, si-SNHG25#1 and si-SNHG25#2 demonstrated relatively higher transfection efficacies (Fig. 2B), and thus, these two siRNAs were used in the subsequent assays. The proliferative ability of EC cells was suppressed upon SNHG25 depletion (Fig. 2C). Additionally, SNHG25 interference induced the apoptosis of EC cells (Fig. 2D). Furthermore, the migration and invasion of EC cells were impeded following si-SNHG25 transfection (Fig. 2E and F). Based on the aforementioned data, it can be assumed that SNHG25 executed tumor-promoting actions in EC cells.

### SNHG25 acts as an miR-497-5p sponge in EC

**Fig. 3:**
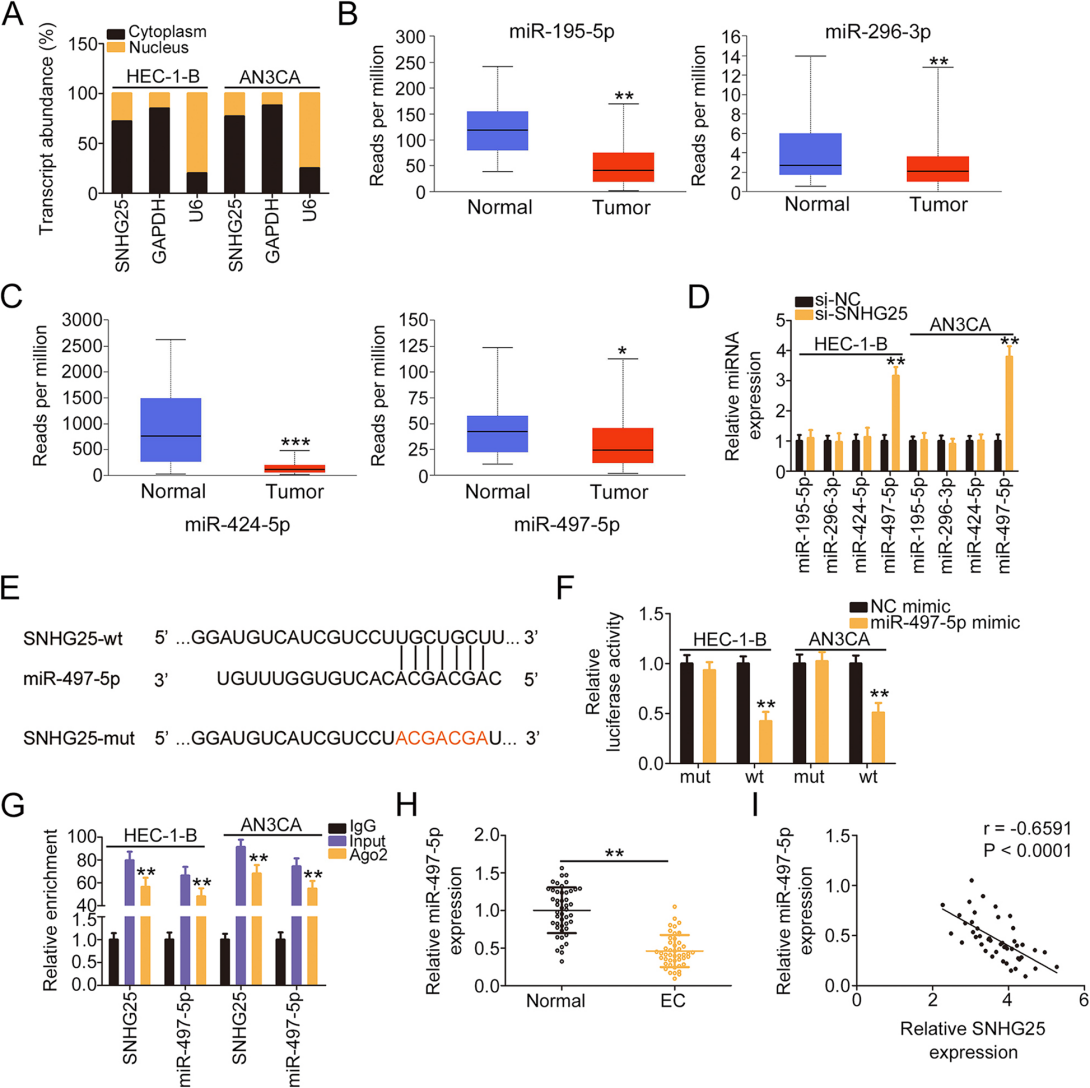
SNHG25 directly sponged iR-497-5p in EC. **A** The subcellular location of SNHG25 in EC cells. **B**, **C** The expression levels of miR-195-5p, miR-296-3p, miR-424-5p, and miR-497-5p in uterine corpus endometrial carcinoma were examined using the TCGA database. **D** The expressions of miR-195-5p, miR-296-3p, miR-424-5p, and miR-497-5p were examined in SNHG25-silenced EC cells. **E** The interacting sequences between SNHG25 and miR-497-5p were predicted via bioinformatic analysis. **F** Luciferase activity was measured in EC cells transfected with SNHG25-wt or SNHG25-mut in parallel with miR-497-5p/NC mimic. **G** RIP assay was used to validate the interaction between SNHG25 and miR-497-5p. **H** qRT-PCR was used for measuring miR-497-5p expression in EC tissues. **I** The correlation between miR-497-5p and SNHG25 expression in EC tissues. **P < 0.01

We next examined the regulatory mechanisms occurring downstream of SNHG25. The subcellular fractionation experiment revealed that SNHG25 was a cytoplasmic lncRNA in EC cells (Fig. 3A); this suggests that it may be a molecular sponge or ceRNA. Moreover, using starBase, 18 miRNAs were observed to contain interacting sites for SNHG25. The levels of the 18 miRNAs were examined using TCGA, which revealed the downregulation of miR-195-5p, miR-296-3p, miR-424-5p, and miR-497-5p in uterine corpus endometrial carcinoma (Fig. 3B and C). Accordingly, they were selected for in-depth analysis. Subsequently, the expression of the four candidates in SNHG25-silenced EC cells was measured. The transfection of si-SNHG25 resulted in the overexpression of miR-497-5p, but miR-195-5p, miR-296-3p, and miR-424-5p expression levels remained unaltered in response to SNHG25 deficiency (Fig. 3D).

The potential binding site of miR-497-5p for SNHG25 is shown in Fig. 3E. The luciferase reporter assay confirmed that ectopic miR-497-5p expression impaired the activity triggered by SNHG25-wt. However, the repressing activity was counteracted once the binding sequences were mutated (Fig. 3F). Furthermore, the RIP assay confirmed that SNHG25 and miR-497-5p were enriched by Ago2 antibody, further implying a direct interaction between them (Fig. 3G). The decrease in miR-497-5p levels in EC tissues was further demonstrated (Fig. 3H), and the existence of an inverse relationship between miR-497-5p and SNHG25 was validated (Fig. 3I). Viewed together, SNHG25 sponges miR-497-5p in EC.

### SNHG25 modulates FASN expression in EC cells by decoying miR-497-5p

**Fig. 4:**
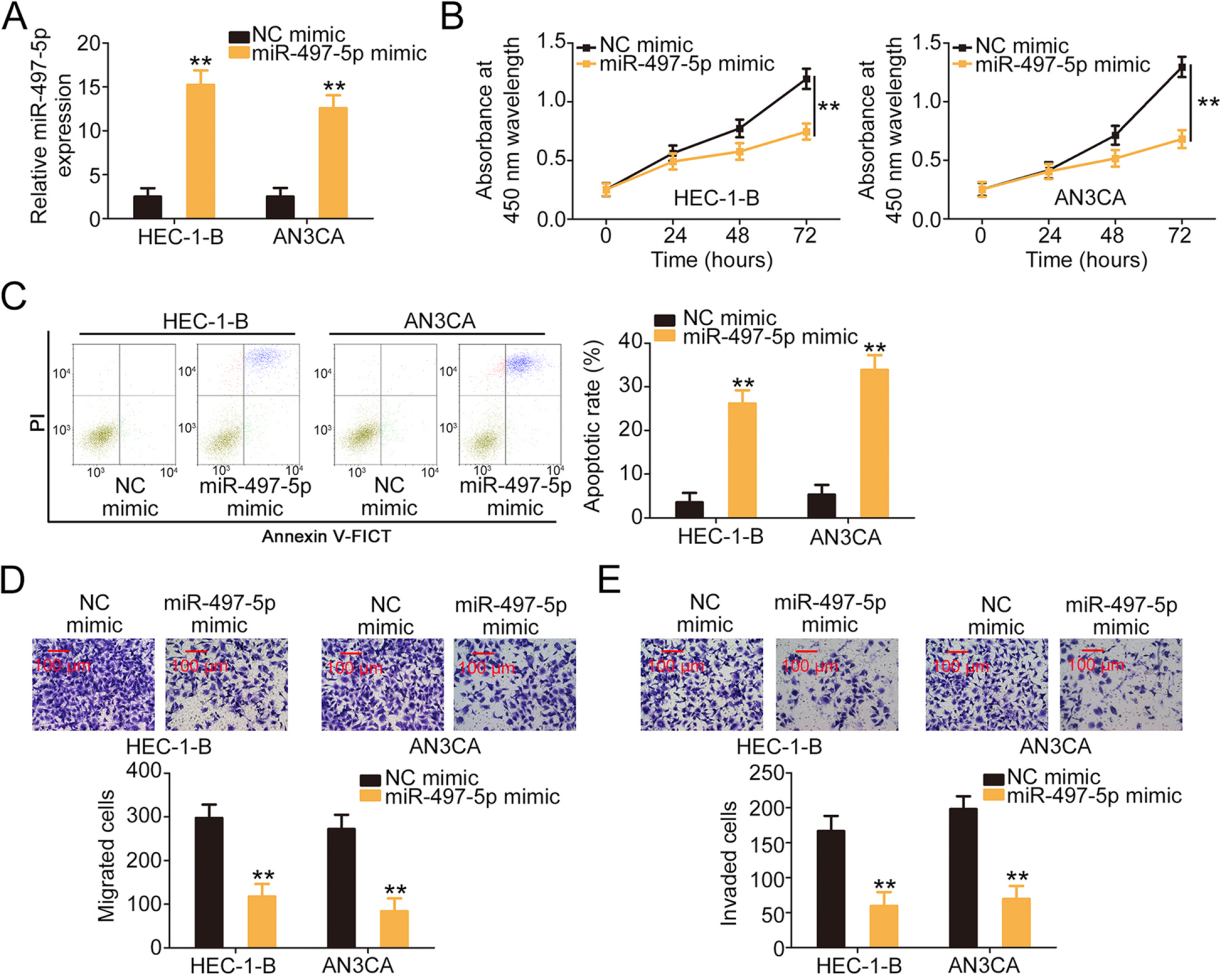
miR-497-5p was confirmed to be a tumor-inhibiting miRNA in EC. **A** The efficiency of miR-497-5p mimic transfection was examined using qRT-PCR. **B**, **C** The proliferation and apoptosis of miR-497-5p-overexpressing EC cells. **D**, **E** Cell migration and invasion of EC cells when the miR-497-5p level was increased. **P < 0.01

The detailed functions of miR-497-5p in EC cells were also clarified. The miR-497-5p expression was markedly increased in EC following transfection with miR-497-5p mimic (Fig. 4A). Cell proliferation was hindered following miR-497-5p overexpression (Fig. 4B). Exogenous miR-497-5p supplementation led to a notable promotion of EC cell apoptosis (Fig. 4C). Moreover, the migratory and invasive properties of EC cells were impaired by miR-497-5p mimic treatment (Fig. 4D and E).

**Fig. 5:**
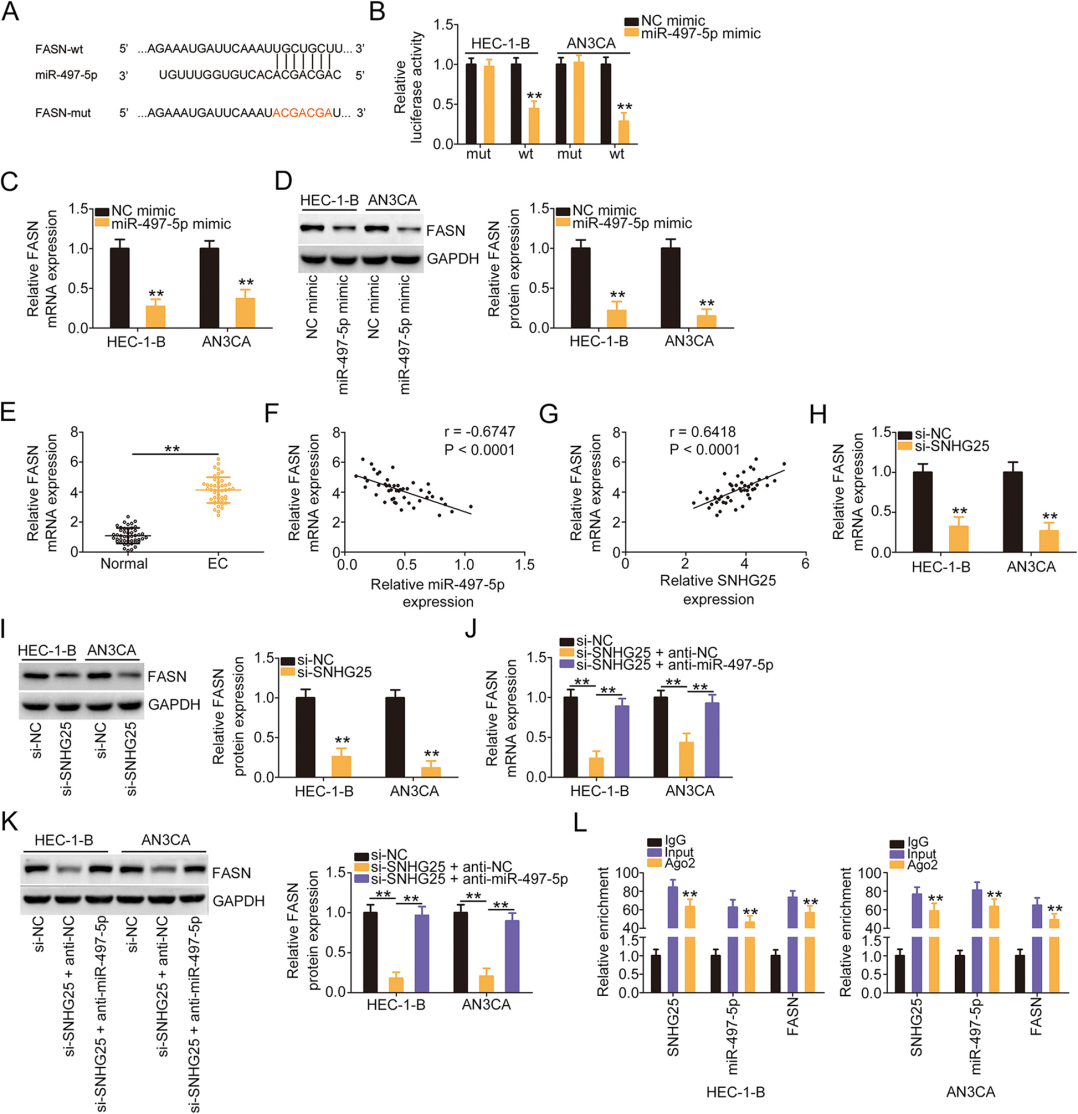
si-SNHG25 lowered FASN level by trapping miR-497-5p. **A** The interacting sequences between miR-497-5p and FASN 3′-UTR. **B** Luciferase activity was tested in EC cells transfected with FASN-wt or FASN-mut in parallel with miR-497-5p/NC mimic. **C**, **D** FASN levels were quantified in EC cells after miR-497-5p upregulation. **E** The expression status of FASN in EC tissues. **F** The expression relationship between FASN and miR-497-5p in EC tissues. **G** The expression relationship between FASN and SNHG25 in EC tissues. **H, I** FASN levels were measured in SNHG25-deficient EC cells. **J**, **K** si-SNHG25, together with anti-miR-497-5p or anti-NC, was transfected into EC cells, followed by the determination of FASN expression. **L** RIP assay was conducted to confirm the enrichment of SNHG25, miR-497-5p, and FASN in immunoprecipitated RNA. **P < 0.01

Bioinformatic prediction revealed a potential binding interaction between miR-497-5p and FASN 3′-UTR (Fig. 5A), which was also validated using the luciferase reporter assay. In contrast to the results observed in the NC mimic group, miR-497-5p mimic suppressed the luciferase activity of FASN-wt, whereas the luciferase activity of FASN-mut remained unaltered in miR-497-5p-overexpressing EC cells (Fig. 5B). Furthermore, FASN expression was downregulated by miR-497-5p mimic in EC cells (Fig. 5C and D). These data prove FASN as a direct target of miR-497-5p in EC.

As aforementioned, SNHG25 sequesters miR-497-5p and miR-497-5p and directly targets FASN in EC. Therefore, we next elucidated whether SNHG25 is involved in the modulation of the miR-497-5p/FASN axis. The expression relationship among them was examined. Overexpressed FASN in EC tissues (Fig. 5E) was negatively associated with miR-497-5p (Fig. 5F) but exhibited a positive relationship with SNHG25 (Fig. 5G). Furthermore, FASN expression was downregulated when SNHG25 was deficient (Fig. 5H and I), which was recovered by anti-miR-497-5p cotransfection (Fig. 5J and K). Finally, the RIP assay revealed that SNHG25, miR-497-5p, and FASN were all enriched by Ago2 antibody in EC cells (Fig. 5L). Thus, SNHG25 functions as a ceRNA for miR-497-5p and consequently increases FASN expression.

### Si-SNHG25 exerts a cancer-repressing role in EC via the adjustment of the miR-497-5p/FASN axis

**Fig. 6:**
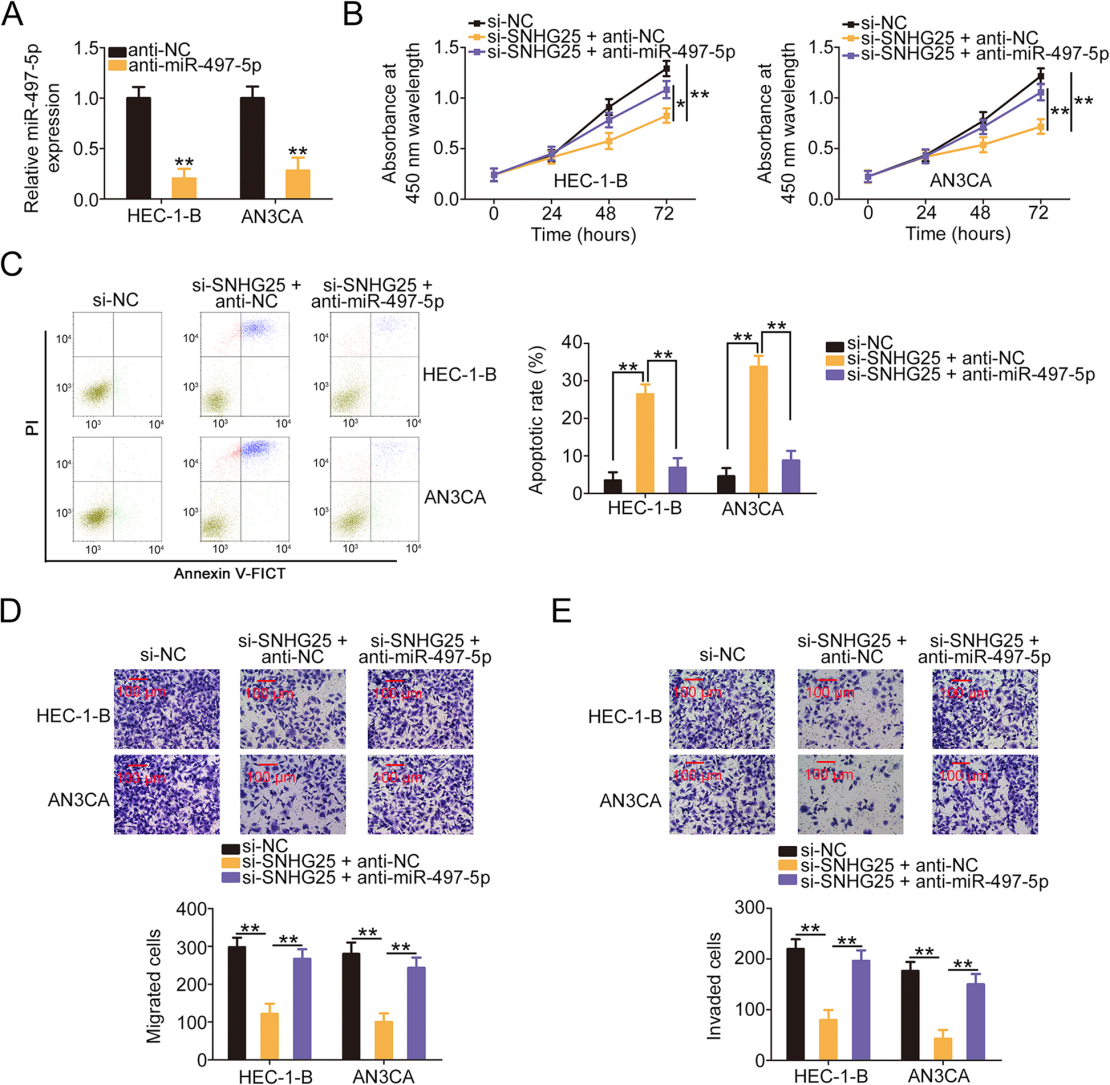
miR-497-5p inhibition abrogated the effects of si-SNHG25 in EC cells. **A**The efficiency of anti-miR-497-5p transfection was examined using qRT-PCR. **B, C** Cell proliferation and apoptosis were assessed in EC cells transfected with si-NC, si-SNHG25 + anti-NC, or si-SNHG25 + anti-miR-497-5p. **D**, **E** The migratory and invasive abilities of EC cells expressing si-NC, si-SNHG25 + anti-NC, or si-SNHG25 + anti-miR-497-5p were examined. *P < 0.05 and **P < 0.01

**Fig. 7:**
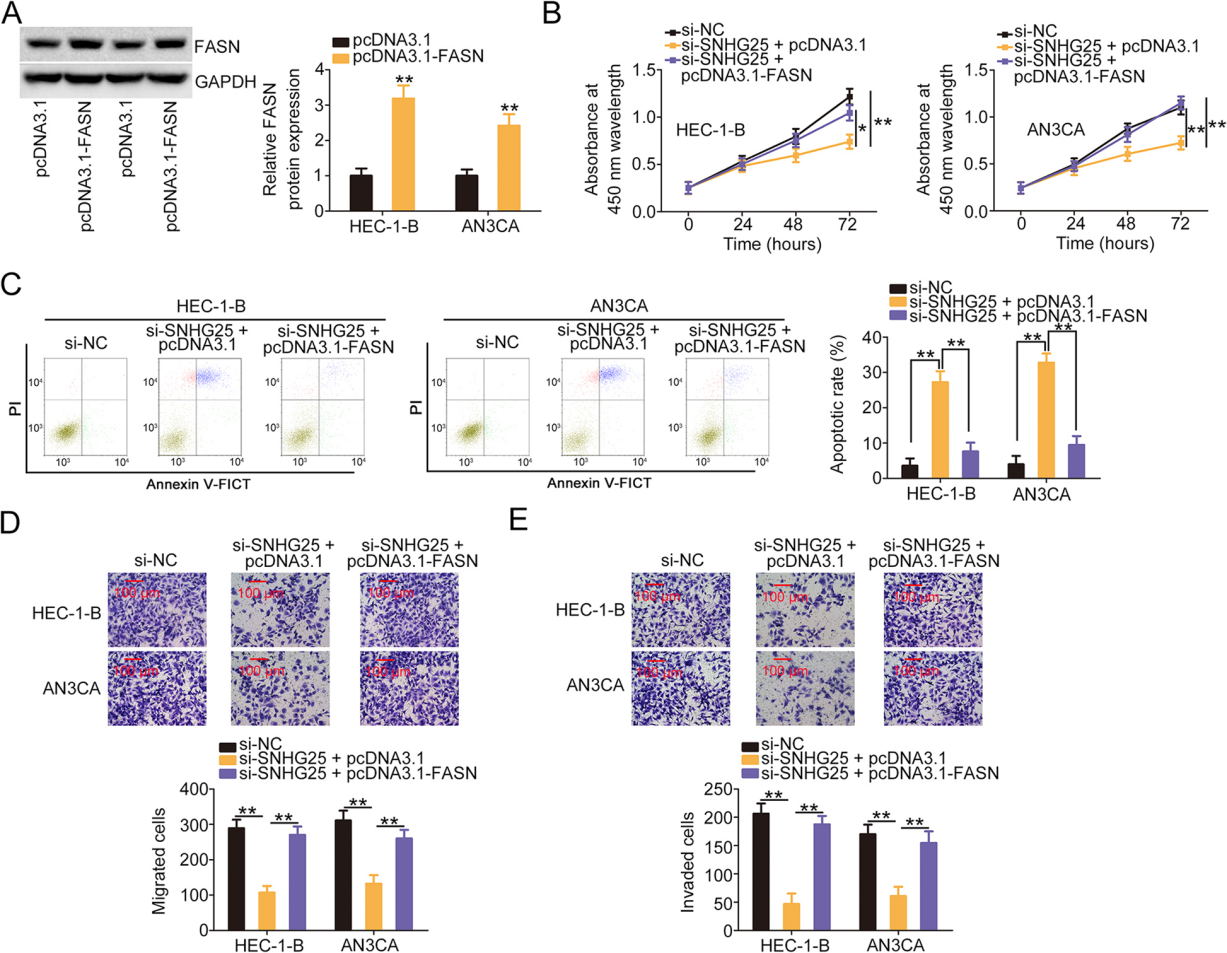
The cancer-repressing effects of si-SNHG25 in EC cells were neutralized by FASN restoration. **A** Western blotting was conducted to validate FASN levels in pcDNA3.1-FASN-transfected EC cells. **B–E** Cell proliferation, apoptosis, and motility were assessed in EC cells transfected with si-NC, si-SNHG25 + pcDNA3.1, or si-SNHG25 + pcDNA3.1-FASN. *P < 0.05 and **P < 0.01

We performed rescue experiments to ascertain whether the regulatory effects of si-SNHG25 in EC cells occurred through the miR-497-5p/FASN axis. The transfection efficiency of anti-miR-497-5p was examined and confirmed by qRT-PCR (Fig. 6A). The proliferation of SNHG25-depleted EC cells significantly declined, and their cell apoptosis was promoted. However, these modulatory actions were abolished by anti-miR-497-5p cotransfection (Fig. 6B and C). Additionally, miR-497-5p downregulation reversed the repressing effects of si-SNHG25 on EC cell migration and invasion (Fig. 6D and E). Furthermore, pcDNA3.1-FASN transfection triggered FASN upregulation (Fig. 7A). SNHG25 downregulation impeded cell proliferation and facilitated cell apoptosis. However, pcDNA3.1-FASN treatment was sufficient to abolish these effects (Fig. 7B and C). Moreover, the migratory and invasive abilities of si-SNHG25-transfected cells were hindered, but these changes were reversed upon FASN reintroduction (Fig. 7D and E). Thus, the miR-497-5p/FASN axis serves as the downstream effector pathway of SNHG25 in EC.

### SNHG25 interference impedes tumor growth in vivo

**Fig. 8:**
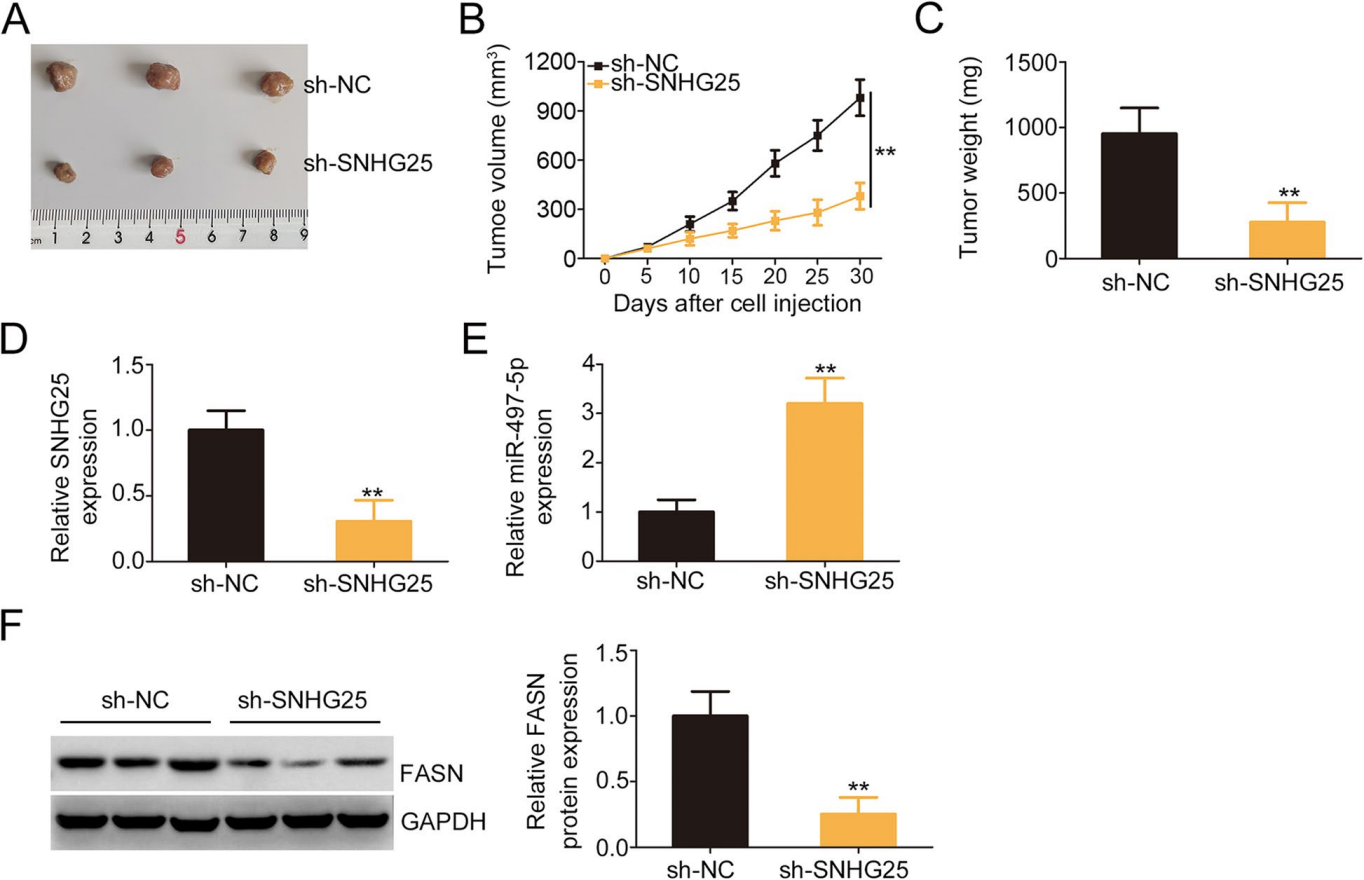
Downregulation of SNHG25 impeded tumor growth in vivo. **A** Representative images of tumor xenografts resected from mice. **B** Tumor size was recorded every 5 days, and the data were used for constructing growth curves. **C** The weight of tumor xenografts was assessed 30 days after treatment. **D, E** Expression of SNHG25 and miR-497-5p in tumor xenografts. **F** The protein level of FASN in tumor xenografts. **P < 0.01

The effect of SNHG25 interference on tumor growth in vivo was examined using tumor xenografts in nude mice. Compared with with the sh-NC group, tumor xenografts from SNHG25-silenced cells were smaller in size (Fig. 8A and B) and lighter in weight (Fig. 8C). Additionally, the downregulation of SNHG25 (Fig. 8D), overexpression of miR-497-5p (Fig. 8E), and reduction of FASN (Fig. 8F) were confirmed in sh-SNHG25-transfected tumor xenografts. Thus, SNHG25 deficiency impedes tumor growth in vivo.

## Discussion

In recent decades, lncRNAs have garnered considerable attention from scholars as a novel research focus [19–21]. The detailed roles of lncRNAs have been intensively studied in EC oncogenesis and progression [22–24]. Several lncRNAs are differentially expressed in EC and involved in controlling numerous aggressive behaviors [25–27]. Despite the fact that the human genome contains over 50,000 lncRNAs [28], their involvement in EC is not completely explored. Herein, we aimed to examine whether SNHG25 plays a key role in EC and investigate its relevant regulatory mechanisms.

SNHG25 is a poorly understood lncRNA. It is upregulated in epithelial ovarian cancer, displaying a significant correlation with the histological grade [18]. SNHG25 overexpression hinders epithelial ovarian cancer cell apoptosis but increases cell proliferation, migration, and invasion [18]. However, whether SNHG25 has clinical role in EC and how it performs its detailed roles remain unknown. Herein, a high level of SNHG25 in EC was confirmed through the TCGA dataset and the cohort of the current study. Patients with a high SNHG25 level had shorter overall survival in contrast to those with a low SNHG25 level. Functionally, SNHG25 deficiency resulted in tumor-repressing activities in EC cells by decreasing cell proliferation, migration, and invasion and promoting cell apoptosis. Furthermore, the function of SNHG25 depletion in impairing tumor growth in vivo was validated. Accordingly, SNHG25 may be a target for diagnosing and treating EC.

The molecular events regulated by lncRNAs are largely dependent on their subcellular location [29]. Nuclear lncRNAs are capable of affecting gene expressions at the transcriptional level via protein interactions [30]. The ceRNA theory is widely actualized by cytoplasmic lncRNAs, wherein gene expression is controlled at the post-transcriptional level [31]. In this context, lncRNAs harbor miRNA response elements, can trap miRNAs, and consequently separate miRNAs from their targets [17]. To examine the mechanisms underlying the actions of SNHG25, we first conducted the subcellular fractionation experiment to elucidate the localization of SNHG25 in EC cells. This revealed that SNHG25 was primarily distributed in EC cell cytoplasm, implying the role of SNHG25 as a ceRNA.

Employing bioinformatic analysis, the complementary binding sequences between SNHG25 and miR-497-5p were predicted. Importantly, this prediction was further validated via multiple laboratory investigations, which revealed that SNHG25 acts as an miR-497-5p sponge. Additionally, it was evinced that miR-497-5p directly targeted FASN in EC. Moreover, miR-497-5p downregulated FASN expression through direct interaction with its 3′-UTR. FASN was under the positive control of SNHG25 in EC cells, which occurred via miR-497-5p sequestration. Notably, as confirmed by RIP, the aforementioned three RNAs were all substantially enriched by Ago2. Thus, the current study provides clear evidence to propose a new ceRNA pathway in EC, involving SNHG25, miR-497-5p, and FASN.

Low miR-497-5p levels have been reported in melanoma [32], non-small-cell lung cancer [33], ovarian cancer [34], and cervical cancer, exerting tumor-repressing activities. Further, miR-497-5p is correlated with EC tumorigenesis [35]. However, the detailed functions of miR-497-5p in EC remain elusive. In this study, miR-497-5p was found to be weakly expressed in EC and executed anticarcinogenic actions during EC oncogenesis and progression. FASN, a central lipogenic enzyme, was proved as a downstream target of miR-497-5p in EC. Overexpression of FASN was associated with multiple aggressive clinicopathological characteristics [36]. FASN served as an important mediator of EC oncogenicity and controlled several biological characteristics [37–39]. The final rescue experiments revealed that the decrease in miR-497-5p or increase in FASN could neutralize the modulatory actions of SNHG25 knockdown in EC cells. Thus, the miR-497-5p/FASN axis operated as the downstream effector pathway through which SNHG25 played cancer-promoting roles in EC.

Our study had several limitations. Firstly, the sample size is small. Secondly, our study only explored the regulatory activities of SNHG25 silencing on EC cells; however, the effects of SNHG25 upregulation on multiple aggressive phenotypes of EC cells were not examined. Furthermore, the mechanisms responsible for the SNHG25 dysregulation in EC were not illustrated. We will resolve these limitations in the near future.

In summary, the current study uncovered that the level of SNHG25 is increased in EC, and depleted SNHG25 impeded EC oncogenicity. Moreover, SNHG25 sequestered miR-497-5p as a ceRNA in EC and consequently positively modulated FASN expression. Therefore, the newly confirmed SNHG25/miR-497-5p/FASN pathway may be useful as a promising target for the molecular-targeted management of EC.

## Data Availability

The analyzed datasets generated during the study are available from the corresponding author on reasonable request.

## Abbreviations

EC: Endometrial cancer
FBS: Fetal bovine serum
NC: Negative control
lncRNA: Long noncoding RNA
miRNA: MicroRNA
SNHG25: Small nucleolar RNA host gene 25
FASN: Fatty acid synthase
